# Association of myosteatosis with short-term outcomes in patients with acute-on-chronic liver failure

**DOI:** 10.1038/s41598-024-64420-x

**Published:** 2024-06-13

**Authors:** Nan Geng, Ming Kong, Jiateng Zhang, Huina Chen, Manman Xu, Wenyan Song, Yu Chen, Zhongping Duan

**Affiliations:** 1 Department of Infectious Diseases, Nanjing Drum Tower Hospital, Affiliated Hospital of Medical School, Nanjing University, Nanjing, 210008 China; 2 Fourth Department of Liver Disease, Beijing Youan Hospital, Capital Medical University, NO.8 Xitou Tiao Road Youwai Street, Beijing, 100069 China; 3 Beijing Municipal Key Laboratory of Liver Failure and Artificial Liver Treatment Research, NO.8 Xitou Tiao Road Youwai Street, Beijing, 100069 China; 4 Beijing Tiantan Hospital, Capital Medical University, Beijing, 100070 China; 5 Department of Clinical Epidemiology and Clinical Trial, Capital Medical University, Beijing, 100069 China; 6 Department of Radiology, Beijing Youan Hospital, Capital Medical University, NO.8 Xitou Tiao Road Youwai Street, Beijing, 100069 China

**Keywords:** Acute on chronic liver failure, Skeletal muscle radiation attenuation, Muscle quality, Sarcopenia, APASL ACLF Research Consortium, Malnutrition, Risk factors

## Abstract

Sarcopenia (low muscle mass, i.e., quantity) is associated with poor clinical outcomes in patients with acute-on-chronic liver failure (ACLF). In this study, we aimed to illustrate the clinical prognostic value of myosteatosis (muscle fat infiltration) for short-term mortality in patients with ACLF. We retrospectively enrolled consecutive patients with ACLF between January 2019 and January 2022. Computed tomography-based body composition analysis was performed at the third lumbar vertebral level to determine skeletal muscle radiation attenuation. Fine and Gray’s competing risk regression model, with liver transplantation as a competing risk, was used to assess the factors associated with 90-day mortality. A total of 431 patients with ACLF were included. Myosteatosis and sarcopenia were observed in 261 (60.6%) and 87 (20.2%) patients, respectively. Competitive risk regression showed that age (HR 1.021, 95% CI 1.000–1.043, *P* = 0.042), APASL ACLF Research Consortium (AARC) score (HR 1.498, 95% CI 1.312–1.710, *P* < 0.001), and sarcopenia (HR 1.802, 95% CI 1.062–3.060, *P* = 0.029) were independently associated with increased 90-day mortality. Subgroup analysis of male patients with HBV-ACLF revealed that myosteatosis (HR 2.119, 95% CI 1.101–4.078, *P* = 0.025) was promising prognostic factors for 90-day mortality after being adjusted for ascites, acute kidney injury, AARC score, and sarcopenia. Myosteatosis is predictive of short-term outcomes in male patients with HBV-ACLF. Our results emphasise the importance of focusing on muscle fat infiltration in patients with HBV-ACLF. Further studies are warranted to investigate the underlying mechanisms and potential therapies for myosteatosis.

## Introduction

In 2010, the European Working Group on Sarcopenia in Older People (EWGSOP) defined sarcopenia as a syndrome characterised by low muscle mass and function (strength or performance)^1^. In 2019, the EWGSOP updated the diagnostic method using low muscle strength, quantity, and quality to confirm sarcopenia. When combined with poor physical performance, this condition is diagnosed as severe sarcopenia^2^. Low muscle quality is an emerging component of skeletal muscle abnormalities that are of equal clinical significance to muscle quantity. One potential cause of the decline in skeletal muscle quality is myosteatosis, characterised by excess muscle fat disposition both in myocytes (intramyocellular fat) and muscle fascia (intermuscular fat)^3^. Recent practice guidelines recommend cross-sectional computed tomography (CT) as the gold standard for quantifying muscle components^4^. Muscle fat accumulation manifests as decreased skeletal muscle radiation attenuation (SM-RA) on CT images of the entire muscle area at the level of the third lumbar vertebra^5^. The inferior clinical implications and predisposing factors of myosteatosis in liver cirrhosis, hepatocellular carcinoma, and post-liver transplantation have been well investigated, and are associated with mortality, infection, hepatic encephalopathy (HE), frailty, and a longer length of hospitalization^6–9^. Some studies have indicated the prognostic superiority of myosteatosis over muscle mass loss alone^10^.

Acute-on-chronic liver failure (ACLF) is a clinical syndrome characterised by acute hepatic decompensation in chronic liver disease with or without cirrhosis and mainly manifests as coagulopathy, jaundice, ascites, and HE^11^. Although a previous study demonstrated the clinical significance of low muscle quantity (sarcopenia) in predicting the short-term prognosis of patients with ACLF^12^, no other studies have explored the prognostic value of low muscle quality (myosteatosis) and the association between myosteatosis and sarcopenia in ACLF patients. Therefore, we investigated the clinical characteristics and prognostic value of myosteatosis in patients with ACLF.

## Materials and methods

### Study population

This was a single center, retrospective cohort study. We consecutively recruited patients with ACLF at Beijing Youan Hospital, Capital Medical University (Beijing, China) between January 2019 and January 2022. The inclusion criteria were as follows: (1) compliance with the diagnostic criteria of ACLF with the Asian-Pacific Association for the Study of the Liver (APASL) consensus, (2) age > 20 years. Exclusion criteria included: (1) liver cancer or other malignant tumours; (2) serious basic diseases of extrahepatic organs, such as chronic renal failure, heart failure, taking anticoagulants after coronary intervention, cerebral haemorrhage or sequelae of cerebral infarction, systemic complications of diabetes and thyroid diseases; (3) complications of other consumptive diseases, such as hyperthyroidism or pulmonary tuberculosis; (4) neuromuscular diseases or long-term bedridden status; (5) those receiving long-term corticosteroid therapy; (6) no available data for height or weight; (7) loss to follow-up; (8) patients who had not undergone CT within 2 weeks of admission.

### Clinical data collection and follow up

Demographic characteristics; pre-existing liver cirrhosis; clinical manifestations upon admission, including ascites, HE, infection, and acute kidney injury (AKI); and baseline laboratory indicators were collected from electronic health records. The diagnosis of liver cirrhosis was based on guidelines on the management of liver cirrhosis developed by Chinese Society of Hepatology^13^.

The above information was extracted independently by two hepatologists (NG and JZ) and discrepancies were resolved by consensus. Patient organ failures (OFs) at admission were evaluated using the European Association for the Study of the Liver-Chronic Liver Failure Consortium (EASL-CLIF C) system. Liver disease severity was determined using the Model for End-Stage Liver Disease-Sodium (MELD-Na) Score, and APASL ACLF Research Consortium (AARC) score. Body mass index (BMI) was calculated as dry weight (kg)/height squared (m^2^), and BMI ≥ 25 kg/m^2^ was defined as overweight. The dry weight for patients with mild, moderate, and bulky ascites was calculated by subtracting 5%, 10%, and 15% of their body weight, respectively. For patients with lower limb edema, we additionally subtracted 5% of their body weight^14^. The degree of ascites and the presence of lower limb edema were evaluated by reviewing abdominal ultrasound reports and electronic medical records. Patients were followed up retrospectively using a medical record system or telephone. The outcomes were death or liver transplantation within 90 days of enrollment.

### Body composition analysis

Abdominal noncontrast-enhanced CT was performed with the patient in the supine position using a 64-row CT scanner (GE LightSpeed). The technical parameters for the CT imaging were as follows: tube voltage, 120 kV; tube current, 380 mA; detector collimation, 0.625 mm; slice thickness, 5 mm; reconstruction thickness, 0.625 mm; and pitch, 5 mm. A single cross-sectional image at the L3 level was analysed by two hepatologists (NG and JZ) using the image analysis software SliceOmatic (V5.0; Tomovision, Magog, Canada). All skeletal muscle areas (SMA) at L3 were identified and quantified using a Hounsfield unit (HU) range of − 29 to 150. The HU thresholds were − 150 to − 50 for visceral adipose tissue area (VATA) and − 190 to − 30 for subcutaneous adipose tissue area (SATA). Cross-sections of the L3-SMA, VATA, and SATA were automatically calculated and normalised for height squared to obtain the skeletal muscle index at the third lumbar vertebra (L3-SMI), visceral adipose tissue index (VATI), and subcutaneous adipose tissue index (SATI) (cm^2^/m^2^). Patients were classified as having sarcopenia using our previously established cut-off values: L3-SMI ≤ 40.2 cm^2^/m^2^ in males and L3-SMI ≤ 31.6 cm^2^/m^2^ in females^15^. Visceral adiposity was defined as VATA > 100 cm^2^^16^. Sarcopenic obesity was defined as a combination of reduced muscle mass and increased BMI (> 25 kg/m^2^) or increased VATA (> 100 cm^2^)^14^. Myosteatosis was defined as SM-RA < 41 Hounsfield unit (HU) in patients with a body mass index (BMI) < 25 kg/m^2^ and SM-RA < 33 HU in patients with a BMI ≥ 25 kg/m^2^^17^.

### Statistical analyses

Continuous variables are presented as mean ± standard deviation (SD) or median (interquartile range) for normally and non-normally distributed data. Categorical data are presented as numbers (percentages). Significant between-group differences were compared using independent sample t-tests or the Mann–Whitney U test. Significant differences between multiple groups were analysed using one-way analysis of variance or the Kruskal–Wallis test coupled with post hoc comparisons. Categorical data were compared using χ^2^ tests. Univariate and multivariate logistic regression analyses were used to identify the risk factors for myosteatosis. The cumulative 90-day mortality according to the presence of myosteatosis were created in the total population as well as subgroup stratified by sex and etiology and compared with gray’s test. Fine and Gray’s competing risk regression model with liver transplantation as a competing risk factor was used to assess the risk factors associated with 90-day mortality. All the images were reviewed by two observers (NG and JZ) to assess inter-observer reliability. To assess intra-observer reliability, images were measured by one observer (NG) twice. Inter- and intra-observer agreements on CT scan readings were determined using the intraclass correlation coefficient (ICC). The percentage of missing value of lactic acid was 8.4%. We performed multiple imputation to impute missing value of lactic acid using the mice R package. Statistical analyses were performed using SPSS (version 24.0, IBM SPSS, Chicago, IL, USA) and R × 64 4.1.2 (http://www.r-project.org/). *P* < 0.05 was considered statistically significant.

### Ethical statement

The retrospective study procedures were approved by the Ethics Committee of Beijing Youan Hospital, Capital Medical University (LL-2020-178-K), and conformed to the Declaration of Helsinki. Informed consent was waived by the Ethics Committee of Beijing Youan Hospital, Capital Medical University because the study was retrospective and the data were anonymized to ensure patient privacy.

## Results

### Study population and baseline characteristics

**Figure 1:**
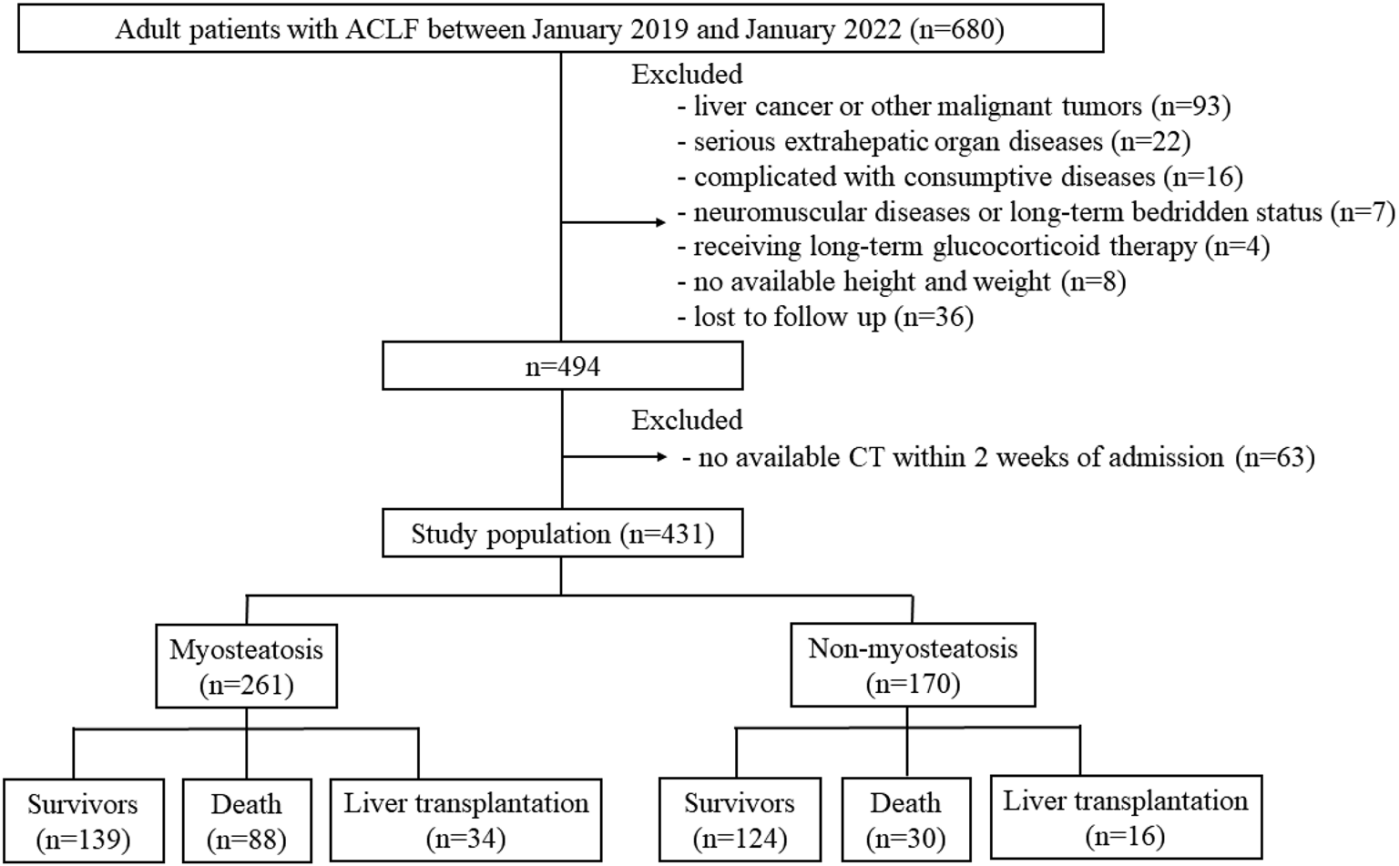
Flow diagram for enrolment in the study.

Among the 680 consecutive adult patients with ACLF between January 2019 and January 2022, 249 patients were excluded. Finally, a total of 431 patients with a mean age of 47 ± 10 years, including 362 (84.0%) male patients were included (Fig. 1). The median BMI was 23.0 (4.8) kg/m^2^, and 128 (29.7) were overweight. The median MELD-Na and AARC scores were 25.0 (9.6) and 9 (2), respectively. Detailed baseline characteristics of the study population are shown in Table 1.

**Table 1:** Baseline characteristics of total cohort and classified by myosteatosis in patients with ACLF.

Variables	Total (n = 431)	Myosteatosis (n = 261)	Non-myosteatosis (n = 170)	*P* value
Age (years)^a^	47 ± 10	50 ± 10	42 ± 9	< 0.001
Males, n (%)^c^	362 (84.0)	198 (75.9)	164 (96.5)	< 0.001
Aetiology				< 0.001
HBV	234 (54.3)	121 (46.4)	113 (66.5)	
Alcohol	109 (25.3)	85 (32.6)	24 (14.1)	
HBV + Alcohol	40 (9.3)	18 (6.9)	22 (12.9)	
Other	48 (11.1)	37 (14.2)	11 (6.5)	
Liver cirrhosis, n (%)^c^	335 (77.7)	214 (82.0)	121 (71.2)	0.008
Ascites, n (%)^c^	342 (79.4)	231 (88.5)	111 (65.3)	< 0.001
HE, n (%)^c^	105 (24.4)	73 (28.0)	32 (18.8)	0.031
AKI, n (%)^c^	71 (16.5)	55 (21.1)	16 (9.4)	0.001
Infection, n (%)^c^	338 (78.4)	214 (82.0)	124 (72.9)	0.026
Diabetes	71 (16.5)	42 (16.1)	29 (17.1)	0.791
MELD-Na score^b^	25.0 (9.6)	26.1 (11.8)	23.9 (7.9)	0.001
AARC score^b^	9 (2)	9 (3)	9 (2)	0.121
Number of OFs n (%)				0.148
0	60 (13.9)	30 (11.5)	30 (17.6)	
1	224 (52.0)	136 (52.1)	88 (51.8)	
≥ 2	147 (34.1)	95 (36.4)	52 (30.6)	
BMI (kg/m^2^)^b^	23.0 (4.8)	22.3 (4.2)	24.8 (5.1)	< 0.001
Overweight, n (%)^c^	128 (29.7)	49 (18.8)	79 (46.5)	< 0.001
L3-SMI (cm^2^/m^2^)^a^	46.5 ± 9.2	43.2 ± 8.4	51.6 ± 7.9	< 0.001
Males	48.1 ± 8.8	45.1 ± 8.6	51.8 ± 7.5	< 0.001
Females	38.0 ± 5.9	37.7 ± 5.9	40.6 ± 6.3	0.258
L3-VATI (cm^2^)^b^	37.5 (35.7)	36.8 (34.1)	38.0 (36.8)	0.884
L3-SATI (cm^2^)^b^	41.4 (31.7)	39.6 (29.6)	44.1 (33.1)	0.317
VSR^b^	0.84 (0.67)	0.87 (0.73)	0.74 (0.60)	0.078
SM-RA^b^	37.0 (9.9)	32.6 (8.2)	42.6 (4.8)	< 0.001
Sarcopenia, n (%)^c^	87 (20.2)	73 (28.0)	14 (8.2)	< 0.001
Sarcopenic obesity, n (%)^c^	40 (9.3)	34 (13.0)	6 (3.5)	0.001
Visceral adiposity, n (%)^c^	236 (54.8)	137 (52.5)	99 (58.2)	0.242

Continuous variables are shown as mean ± standard deviation or median (interquartile range) for normally and non-normally distributed continuous variables, respectively. Categorical variables are presented as numbers (percentages).

^a^Independent sample t-test, ^b^Mann–Whitney U test, or ^c^χ^2^ test was used to compare two groups of continuous or categorical variables.

### Clinical characteristics and influencing factors of myosteatosis in patients with ACLF

The median time between CT imaging used for segmentation and admission was 3 days (IQR 1–5). High intra-observer (ICC = 0.996, *P* < 0.001) and inter-observer agreement (ICC = 0.994, *P* < 0.001) were observed. The mean L3-SMI of enrolled participants was 46.5 ± 9.2 cm^2^/m^2^, and 87 (20.2%) patients were categorised with sarcopenia, based on our pre-defined cutoff values. The median VATI, SATI, VSR, and SM-RA at L3 were 37.5 (35.7) cm^2^/m^2^, 41.4 (31.7) cm^2^/m^2^, 0.84 (0.67), and 37.0 (9.9) HU, respectively. Visceral adiposity, sarcopenic obesity, and myosteatosis were observed in 236 (54.8%), 40 (9.3%), and 261 (60.6%) patients, respectively. Compared to patients without myosteatosis, patients with myosteatosis were older (49.6 ± 10.0 vs 42.2 ± 8.9, *P* < 0.001) and had significantly increased MELD-Na scores (26.1 [11.8] vs 23.9 [7.9], *P* = 0.001). Patients with myosteatosis had a higher incidence of ascites (88.5% vs 65.3%, *P* < 0.001), HE (28% vs 18.8%, *P* = 0.031), AKI (21.1% vs 9.4%, *P* = 0.001), infection (82% vs 72.9%, *P* = 0.026), sarcopenia (28% vs 8.2%, *P* < 0.001), and sarcopenic obesity (13.0% vs 3.5%, *P* < 0.001) compared with non-myosteatosis patients (Table 1). As shown in Table 2, in the multivariate logistic regression, advanced age (odds ratio [OR] 1.074, 95% CI 1.046–1.102, *P* < 0.001), female sex (OR 12.648, 95% CI 4.242–37.712, *P* < 0.001), alcoholic liver disease (OR 3.098, 95% CI 1.676–5.725, *P* < 0.001), and sarcopenia (OR 4.002, 95% CI 1.595–10.046, *P* = 0.003) were independent risk factors of myosteatosis in patients with ACLF.

**Table 2:** Factors associated with myosteatosis according to univariate and multivariate logistic analysis in patients with ACLF.

Variables	Univariate analysis		Multivariate analysis	
	OR (95% CI)	*p*-value	OR (95% CI)	*p*-value
Age	1.084 (1.059–1.108)	< 0.001	1.074 (1.046–1.102)	< 0.001
Sex				
Males	Ref		Ref	
Females	8.697 (3.671–20.605)	< 0.001	12.648 (4.242–37.712)	< 0.001
Aetiology				
HBV	Ref		Ref	
Alcohol	3.308 (1.965–5.567)	< 0.001	3.098 (1.676–5.725)	< 0.001
HBV + Alcohol	0.764 (0.390–1.499)	0.434	0.951 (0.453–1.997)	0.894
Other	3.141 (1.529–6.455)	0.002	0.422 (0.150–1.185)	0.101
Liver cirrhosis	1.844 (1.166–2.915)	0.009	1.541 (0.899–2.643)	0.116
Diabetes	0.932 (0.555–1.566)	0.791		
Sarcopenia	4.327 (2.351–7.963)	< 0.001	4.002 (1.595–10.046)	0.003
VSR	1.440 (1.009–2.056)	0.045	0.987 (0.602–1.619)	0.959
VATI	1.003 (0.995–1.011)	0.456		
SATI	1.000 (0.993–1.007)	0.957		
Visceral obesity	0.792 (0.537–1.170)	0.242		
Sarcopenic obesity	4.094 (1.680–9.978)	0.002	1.041 (0.284–3.810)	0.952

Age, sex, aetiology, liver cirrhosis, sarcopenia, VSR, and sarcopenic obesity were included in the multivariate logistic analysis.

### Association of myosteatosis with 90-day mortality in the total cohort

After 90-days of follow-up, 118 (27.4) patients died, 50 (11.6) underwent liver transplantation, and 263 (61.0) survived without liver transplantation. As shown in Supplementary Fig. 1, the 90-day cumulative mortality in patients with myosteatosis was significantly higher than that in patients without myosteatosis. We further performed univariate and multivariate competing risk regressions to assess independent factors associated with 90-day mortality (Table 3). In the multivariate analysis, age (hazard ratio [HR] 1.021, 95% CI 1.000–1.043, *P* = 0.042), AARC score (HR 1.498, 95% CI 1.312–1.710, *P* < 0.001), and sarcopenia (HR 1.802, 95% CI 1.062–3.060, *P* = 0.029) were independent risk factors for increased 90-day mortality, but this was not the case among patients with myosteatosis (HR, 1.477, 95% CI 0.900–2.424, *P* = 0.123).

**Table 3:** Univariate and multivariate competing risk regression for predicting 90-day mortality in the total cohort.

Variables	Univariate analysis		Multivariate analysis	
	HR (95% CI)	*P-*value	HR (95% CI)	*P-*value
Age	1.022 (1.004–1.040)	0.045	1.021 (1.000–1.043)	0.042
Liver cirrhosis	0.942 (0.599–1.481)	0.796		
Ascites	2.096 (1.197–3.670)	0.010	0.999 (0.559–1.784)	0.996
AKI	2.500 (1.675–3.732)	< 0.001	1.231 (0.760–1.996)	0.398
Infection	2.056 (1.196–3.534)	0.009	1.321 (0.775–2.310)	0.329
Diabetes	1.438 (0.929–2.226)	0.103		
AARC score	1.521 (1.356–1.706)	< 0.001	1.498 (1.312–1.710)	< 0.001
Overweight	0.692 (0.447–1.072)	0.099		
Sarcopenia	2.219 (1.533–3.212)	< 0.001	1.802 (1.062–3.060)	0.029
Visceral obesity	0.752 (0.525–1.078)	0.121		
Myosteatosis	2.076 (1.369–3.149)	0.001	1.477 (0.900–2.424)	0.123
Sarcopenic obesity	2.000 (1.268–3.154)	0.003	0.786 (0.412–1.500)	0.466

Age, ascites, AKI, infection, AARC score, sarcopenia, myosteatosis, and sarcopenic obesity were included in the multivariate analysis.

### Prognostic role of myosteatosis in male patients with HBV-ACLF

**Figure 2:**
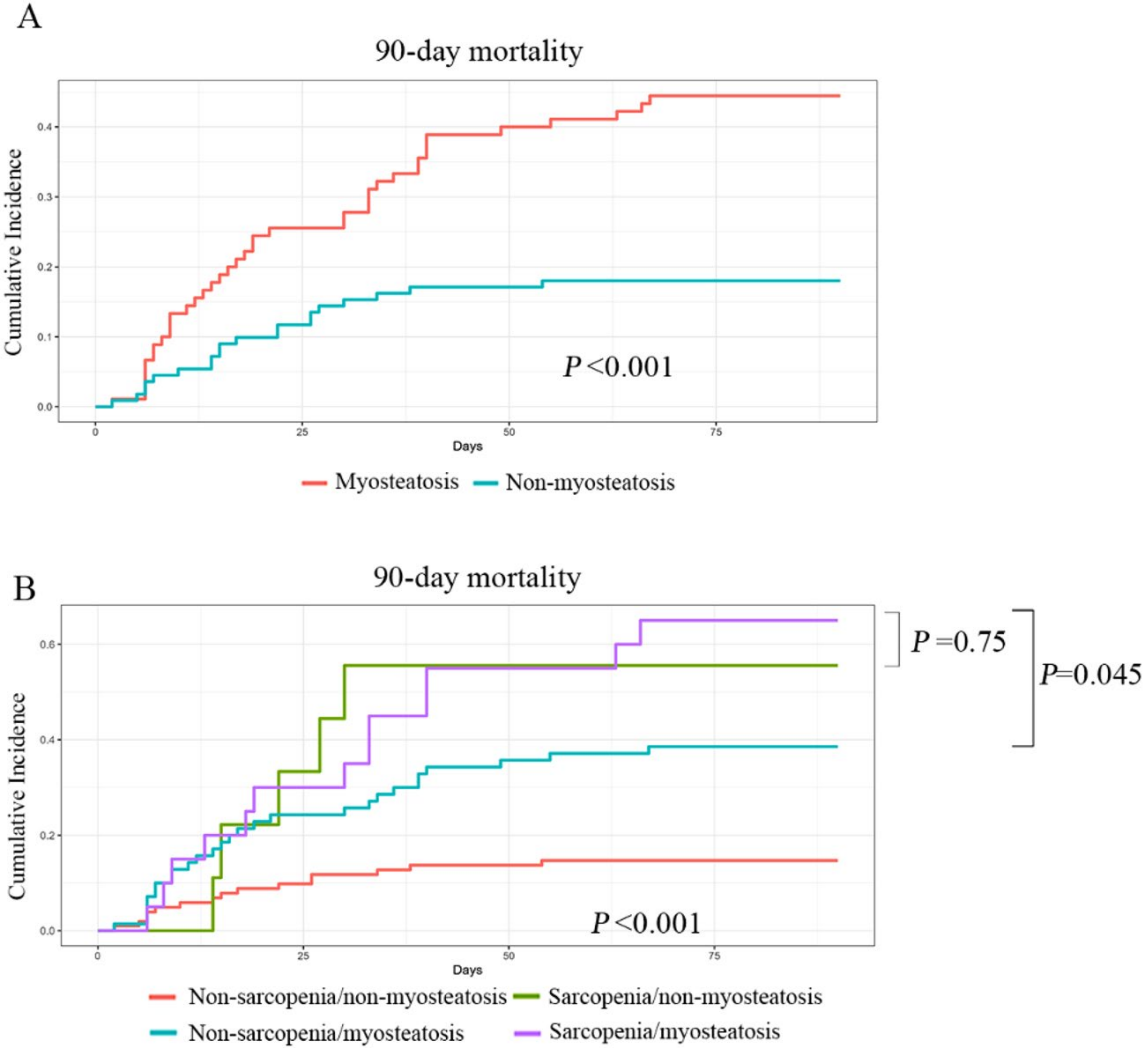
The 90-day cumulative mortality in the groups with and without myosteatosis (**A**), and the groups with co-existing sarcopenia and myosteatosis in male patients with HBV-ACLF (**B**).

Female sex and alcoholic liver disease were independent risk factors for myosteatosis. As a continuous or categorical variable, SM-RA level and incidence rate of myosteatosis varied significantly among aetiologies and sexes (Supplementary Fig. 2). Therefore, incorporating different aetiologies and sex into the analysis may have confounding effects. In Asia, hepatitis B is the main cause of ACLF, and males account for over 80% of ACLF cases. Therefore, we investigated the 90-day prognostic value of myosteatosis in male patients with HBV-ACLF. During the 90-day follow-up of 201 male patients with HBV-ACLF, 60 (29.9%) died, 30 (14.9%) underwent liver transplantation, and 111 (55.2%) were alive without liver transplantation. The 90-day cumulative mortality was significantly higher in patients with myosteatosis than in those without myosteatosis (Fig. 2A). We further performed univariate and multivariate competing risk regression analyses to analyse independent risk factors for 90-day mortality. Factors with *P* < 0.01 in the univariate analysis were further included in the multivariate analysis. AARC scores (HR 1.583, 95% CI 1.308–1.916, *P* < 0.001), sarcopenia (HR 2.025, 95% CI 1.061–3.862, *P* = 0.025) and myosteatosis (HR 2.119, 95% CI 1.101–4.078, *P* = 0.025) were independently associated with 90-day mortality in male patients with HBV-ACLF in the multivariate regression analysis (Table 4).

**Table 4:** Univariate and multivariate competing risk regression for predicting 90-day mortality in male patients with HBV-ACLF.

Variables	Univariate analysis		Multivariate analysis	
	HR (95% CI)	*P* value	HR (95% CI)	*P* value
Age	1.024 (0.999–1.049)	0.061		
Overweight	0.629 (0.345–1.144)	0.129		
Liver cirrhosis	0.978 (0.540–1.770)	0.940		
Ascites	4.307 (1.737–10.678)	0.002	1.327 (0.449–3.920)	0.608
AKI	3.766 (1.850–7.667)	< 0.001	1.127 (0.452–2.809)	0.797
Infection	2.406 (1.107–5.231)	0.027		
AARC score	1.613 (1.363–1.909)	< 0.001	1.583 (1.308–1.916)	< 0.001
Sarcopenia	3.027 (1.806–5.076)	< 0.001	2.025 (1.061–3.862)	0.025
Visceral obesity	0.528 (0.317–0.878)	0.014		
Myosteatosis	2.832 (1.653–4.854)	< 0.001	2.119 (1.101–4.078)	0.025
Sarcopenic obesity	2.122 (1.032–4.364)	0.041		

Ascites, AKI, AARC score, sarcopenia, and myosteatosis were concluded in the multivariate analysis.

### The role of the concomitant presence of myosteatosis and sarcopenia in male patients with HBV-ACLF

Among the 201 male patients with HBV-ACLF, 102 (50.7%) had neither sarcopenia nor myosteatosis (NN), nine (4.5%) had sarcopenia alone (SN), 70 (34.8%) had myosteatosis alone (NM), and only 20 (10.0%) had comorbid sarcopenia and myosteatosis (SM). The baseline characteristics of the male HBV-ACLF cohort classified according to sarcopenia and myosteatosis are shown in Supplementary Table 1. The 90-day cumulative mortality in patients with coexisting sarcopenia and myosteatosis was higher than that of myosteatosis alone (*P* = 0.045), but not different from that in patients with sarcopenia alone (*P* = 0.75), as shown in Fig. 2B.

## Discussion

Sarcopenia was significantly associated with the 90-day mortality in patients with all-cause ACLF and subgroup analysis of males with HBV-ACLF. Sarcopenia has become a popular topic in recent years, and its importance is being recognized by more and more disciplines. However, according to our diagnostic criteria for sarcopenia, its incidence rate was low, 20.2% in the total cohort, and only 14.4% in male patients with HBV-ACLF. Compared with sarcopenia, there are fewer studies on myosteatosis, which is an emerging component of skeletal muscle abnormalities. To the best of our knowledge, this is the first study to evaluate the association between myosteatosis and short-term outcomes in patients with ACLF. Notably, the subgroup analysis of male patients with HBV-ACLF showed a strong association between myosteatosis and increased 90-day mortality rate. These results support the importance of focusing on muscle quality in patients with HBV-ACLF.

Various diagnostic criteria can characterise myosteatosis, depending on reference populations and outcomes, without a clear consensus. Many studies have diagnosed myosteatosis based on a lower SM-RA measured in HU on CT, which represents an increased proportion of intramuscular adipose tissue and intramyocellular lipids. The widely used cutoff values of < 33 HU in patients with BMI ≥ 25 kg/m^2^ and < 41 for BMI < 25 kg/m^2^ were derived from a cancer population^17^. Additionally, muscle quality evaluation by CT at L3 using the intramuscular adipose tissue content (IMAC) parameter was initially first proposed to explore the association of IMAC with non-alcoholic steatohepatitis severity, which is calculated by the attenuation of the multifidus muscles/subcutaneous fat tissue attenuation ratio^18^. Higher IMAC indicates abundant fat deposition in muscle tissue, and thus, lower muscle quality. Recently, Meister et al*.* performed an analysis of 264 patients who underwent orthotopic liver transplantation (OLT) and compared the prognostic role of three frequent myosteatosis diagnosis criteria (quartile-based cutoff values for L3Muscle-RA, L3Psoas-RA, and L3-IMAC). L3Muscle-RA performed better than L3Psoas-RA and L3-IMAC in predicting adverse short-term outcomes after deceased-donor OLT^19^. In summary, we adopted the most widely used method to diagnose myosteatosis.

The mechanism contributing to excessive lipid accumulation within skeletal muscles is not completely understood, but may be related to metabolic abnormalities and mitochondrial dysfunction^3^. Excess lipids "overflow" from adipocytes and redistribute to other tissues, especially skeletal muscles^20^. In the early phase of lipid overload, oxidative muscles increase-oxidation capacity to inhibit excessive lipid deposition in muscle cells. Following excessive exposure to lipids, fatty acid oxidation decreases, and lipids are deposited due to mitochondrial dysfunction of the oxidative muscle. Body composition is characterised by sex-specific differences in muscle homeostasis and metabolism. We previously demonstrated that in healthy individuals, skeletal muscle mass and visceral adipose tissue in males are significantly higher than that in females, while subcutaneous adipose tissue in females is significantly higher than that in males^15,16^. In the present study, female patients had lower SM-RA levels and were more likely to develop myosteatosis. Differences in muscle types and sex differences in muscle metabolism are factors that must be considered when studying myosteatosis^21^. Liver disease may have different effects on skeletal muscle metabolism. In this study, we confirmed a strong association between alcoholic liver disease and myosteatosis. Ethanol can impair muscle protein homeostasis, promote fat accumulation, and increase the sensitivity of skeletal muscles to hyperammonaemia. Moreover, patients with non-alcoholic fatty experience increased fatty acid and insulin resistance, potentially inducing additional adverse effects, such as metabolic dysregulation^22^. Considering the significant differences between sexes and aetiologies, myosteatosis should be studied in sex- and aetiology-specific populations.

Muscle fat infiltration may not occur simultaneously with the loss of muscle mass. Whether Muscle fat infiltration is caused by the loss of muscle mass remains unclear, and the association between myosteatosis and sarcopenia remains controversial. Notably, our results showed that sarcopenia is closely related to myosteatosis. Similarly, a study conducted including 362 patients with chronic liver disease demonstrated that older age, female sex, presence of sarcopenia, and higher VSR levels were significant independent factors associated with myosteatosis^23^. In contrast, a retrospective analysis of 473 patients with decompensated cirrhosis revealed that advanced age, higher VSR, and higher VATI were independently associated with myosteatosis; however, there was no interaction between sarcopenia and myosteatosis^24^. This discrepancy may be attributed to the different thresholds and methods used to identify myosteatosis. We speculate that the excessive accumulation of lipids within skeletal muscles can lead to a decrease in muscle mass and dysfunction, and vice versa. The probable mechanisms are as follows: (1) myosteatosis may induce skeletal muscle mitochondrial dysfunction, insulin resistance, differentiation of muscle stem cells into adipocytes, and lipotoxicity, which inhibits protein synthesis and leads to decreased muscle mass and function. (2) sarcopenia exacerbates lipid accumulation in skeletal muscles due to small oxidised muscle fibres and the inability to effectively carry out fatty acid mitochondria β Oxidation^3,25^.

The association between myosteatosis and mortality has been evaluated for several medical conditions (Supplementary Table 2). Myosteatosis is negatively correlated with clinical prognosis, particularly when predicting short-term outcomes. Recently Czigany et al.^10^ investigated the role of myosteatosis in predicting perioperative outcomes in deceased donor OLT. In the first 3 months after OLT, patients with myosteatosis had more severe surgical complications, increased need for intraoperative blood transfusions, increased rates of early allograft dysfunction, higher comprehensive complication index scores, longer ICU and hospital stays, and higher procedural costs. Another study by the same group investigated the effects of myosteatosis on long-term graft and patient survival after OLT and multivariable analysis revealed that myosteatosis was an independent predictor of adverse 5-year survival. However, the significant effect of myosteatosis was lost in univariate and multivariate analyses, from which patients who died within the first 90 days after OLT were excluded^8^. These findings indicate that myosteatosis may be a good predictor of the short-term prognosis of patients with end-stage liver disease. The additive effects of sarcopenia and myosteatosis on poor prognosis have been well studied. For example, concomitant sarcopenia and myosteatosis are associated with worse survival than the respective conditions alone^26^. In our study, myosteatosis plays an important prognostic role in male patients with HBV-ACLF. Notably, the 90-day cumulative mortality in patients with coexisting sarcopenia and myosteatosis was higher than that of myosteatosis alone. Therefore, for patients with myosteatosis, it is essential to provide enhanced care immediately when they are on the waiting list.

The cause of death for the patient with ACLF may be attributed to severe infections and multiple organ failures, including liver, kidneys, brain, respiratory and circulatory systems. The high mortality in ACLF patients with myosteatosis may be related to the high incidence of infections, impaired liver regeneration and systemic inflammatory response. Infection is the main complication of end-stage liver disease. A recent study demonstrated that the high risk of mortality associated with myosteatosis was associated with a higher frequency of sepsis-related deaths in patients with cirrhosis^27^. Excess adipose accumulation in skeletal muscles may affect muscle fibre orientation and is associated with decreased muscle strength, physical performance, and reduced resistance to stressors. Timely liver regeneration depends on adequate availability of energy and metabolites, and a decline in body reserves may reduce liver regeneration ability^28^. Additionally, myosteatosis is associated with increased inflammatory cytokine release and abnormal myokine and adipokine secretion, which may activate and recruit macrophages by binding to chemokine receptors or toll-like receptor 4, leading to an inflammatory cascade^25^. Ectopic fat can induce the formation of NLRP3 inflammasomes, thereby activating caspase1 and subsequently IL-1β and IL-18, and exacerbating systemic inflammation^29^.

Our study had several limitations. First, this was a retrospective, single-center study. Further multicenter prospective studies are imperative to confirm the generalizability and applicability of our findings. Second, we analysed only the prognostic role of myosteatosis at baseline. It is unclear whether ACLF is an aggravating factor of myosteatosis and whether the dynamic decline in muscle quality is more meaningful in predicting the prognosis. Third, although we used the most widely used method to diagnose myosteatosis, the suitability of these cutoff points for our population remains to be verified. Lastly, we hypothesized that inflammation, and impaired liver regeneration are the reasons for the poor prognosis of myosteatosis; however, comprehensive inflammatory cytokines and liver regeneration indices were not available due to the retrospective design of our study. The pathophysiological mechanisms underlying the poor clinical outcomes of ACLF with myosteatosis require further study. Therefore, there is an urgent need to investigate the pathogenesis and treatment methods of myosteatosis, establish appropriate diagnostic methods, and verify our conclusions in a prospective multicentre cohort study.

## Conclusion

Our findings indicate that myosteatosis is independently associated with advanced age, female sex, alcoholic liver disease, and sarcopenia and is considered an independent predictor of short-term outcomes in male patients with HBV-ACLF. Myosteatosis may serve not only as a prognostic factor but also as a therapeutic target. The study emphasises the importance of identifying individuals muscle fat infiltration among those with normal muscle mass in clinical settings and may lay a foundation for clinician to improve patient care and plan the optimal treatment. Further studies are warranted to investigate the pathophysiology and therapeutic strategies for myosteatosis.

### Supplementary Information

Supplementary Information.

## Data Availability

Our data is available upon request. Please contact the correspondence author for access to the data.

## References

[1] Cruz-Jentoft AJ, Baeyens JP, Bauer JM (2010). Sarcopenia: European consensus on definition and diagnosis: Report of the European Working Group on Sarcopenia in Older People. Age Ageing.

[2] Cruz-Jentoft AJ, Bahat G, Bauer J (2019). Sarcopenia: Revised European consensus on definition and diagnosis. Age Ageing.

[3] Ebadi M, Tsien C, Bhanji RA (2022). Myosteatosis in cirrhosis: A review of diagnosis, pathophysiological mechanisms and potential interventions. Cells.

[4] Lai JC, Tandon P, Bernal W (2021). Malnutrition, frailty, and sarcopenia in patients with cirrhosis: 2021 practice guidance by the american association for the study of liver diseases. Hepatology.

[5] Reichelt S, Pratschke J, Engelmann C (2022). Body composition and the skeletal muscle compartment in liver transplantation: Turning challenges into opportunities. Am. J. Transplant.

[6] Bhanji RA, Moctezuma-Velazquez C, Duarte-Rojo A (2018). Myosteatosis and sarcopenia are associated with hepatic encephalopathy in patients with cirrhosis. Hepatol. Int..

[7] Feng H, Wang X, Mao L (2021). Relationship between sarcopenia/myosteatosis and frailty in hospitalized patients with cirrhosis: A sex-stratified analysis. Ther. Adv. Chronic. Dis..

[8] Czigany Z, Kramp W, Lurje I (2021). The role of recipient myosteatosis in graft and patient survival after deceased donor liver transplantation. J. Cachexia Sarcopenia Muscle.

[9] Nardelli S, Lattanzi B, Merli M (2019). Muscle alterations are associated with minimal and overt hepatic encephalopathy in patients with liver cirrhosis. Hepatology.

[10] Czigany Z, Kramp W, Bednarsch J (2020). Myosteatosis to predict inferior perioperative outcome in patients undergoing orthotopic liver transplantation. Am. J. Transplant.

[11] Sarin SK, Choudhury A, Sharma MK (2019). Acute-on-chronic liver failure: consensus recommendations of the Asian Pacific association for the study of the liver (APASL): An update. Hepatol. Int..

[12] Peng H, Zhang Q, Luo L (2022). A prognostic model of acute-on-chronic liver failure based on sarcopenia. Hepatol. Int..

[13] Xu X-Y, Ding H-G, Li W-G (2020). Chinese guidelines on the management of liver cirrhosis (abbreviated version). World J. Gastroenterol..

[14] Feng H, Wang X, Zhao T (2021). Myopenic obesity determined by visceral fat area strongly predicts long-term mortality in cirrhosis. Clin. Nutr..

[15] Kong M, Geng N, Zhou Y (2022). Defining reference values for low skeletal muscle index at the L3 vertebra level based on computed tomography in healthy adults: A multicentre study. Clin. Nutr..

[16] Kong M, Xu M, Zhou Y (2022). Assessing visceral obesity and abdominal adipose tissue distribution in healthy populations based on computed tomography: A large multicenter cross-sectional study. Front. Nutr..

[17] Martin L, Birdsell L, Macdonald N (2013). Cancer cachexia in the age of obesity: Skeletal muscle depletion is a powerful prognostic factor, independent of body mass index. J. Clin. Oncol..

[18] Kitajima Y, Hyogo H, Sumida Y (2013). Severity of non-alcoholic steatohepatitis is associated with substitution of adipose tissue in skeletal muscle. J. Gastroenterol. Hepatol..

[19] Meister FA, Bednarsch J, Amygdalos I (2021). Various myosteatosis selection criteria and their value in the assessment of short- and long-term outcomes following liver transplantation. Sci. Rep..

[20] Ritter O, Jelenik T, Roden M (2015). Lipid-mediated muscle insulin resistance: Different fat, different pathways?. J. Mol. Med. (Berl).

[21] Della Peruta C, Lozanoska-Ochser B, Renzini A (2023). Sex differences in inflammation and muscle wasting in aging and disease. Int. J. Mol. Sci..

[22] Welch N, Dasarathy J, Runkana A (2020). Continued muscle loss increases mortality in cirrhosis: Impact of aetiology of liver disease. Liver Int..

[23] Tachi Y, Kozuka A, Hirai T (2018). Impact of myosteatosis on skeletal muscle volume loss in patients with chronic liver disease. J. Gastroenterol. Hepatol..

[24] Wang X, Sun M, Li Y (2022). Association of myosteatosis with various body composition abnormalities and longer length of hospitalization in patients with decompensated cirrhosis. Front. Nutr..

[25] Li CW, Yu K, Shyh-Chang N (2022). Pathogenesis of sarcopenia and the relationship with fat mass: Descriptive review. J. Cachexia Sarcopenia Muscle.

[26] Ebadi M, Tsien C, Bhanji RA (2022). Skeletal muscle pathological fat infiltration (myosteatosis) is associated with higher mortality in patients with cirrhosis. Cells.

[27] Montano-Loza AJ, Angulo P, Meza-Junco J (2016). Sarcopenic obesity and myosteatosis are associated with higher mortality in patients with cirrhosis. J. Cachexia Sarcop. Muscle.

[28] Uluk D, Pratschke J, Lurje G (2021). Influence of skeletal muscle mass on graft regeneration after living-donor liver transplantation. Hepatobiliary Surg. Nutr..

[29] Vandanmagsar B, Youm Y-H, Ravussin A (2011). The NLRP3 inflammasome instigates obesity-induced inflammation and insulin resistance. Nat. Med..

