# Supplementary material for: Association of myosteatosis with short-term outcomes in patients with acute-on-chronic liver failure
Source: Sci Rep. 2024 Jun 13;14:13609. doi: 10.1038/s41598-024-64420-x (PMC11176162; doi:10.1038/s41598-024-64420-x)
Supplement: Supplementary file 1 — Supplementary Information. [file 41598_2024_64420_MOESM1_ESM.docx]

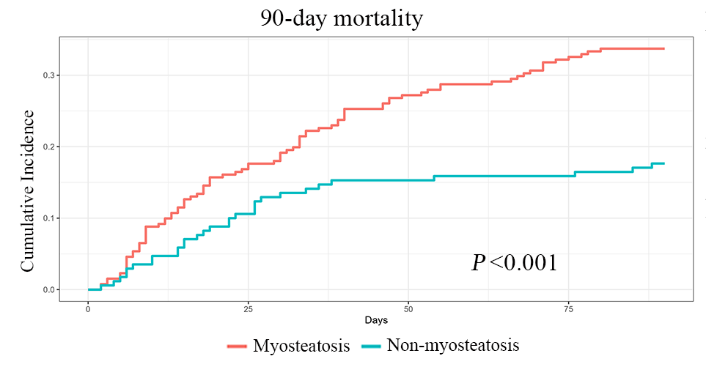


Supplementary Figure 1. The 90-day cumulative mortality according to myosteatosis and non-myosteatosis in the total cohort.


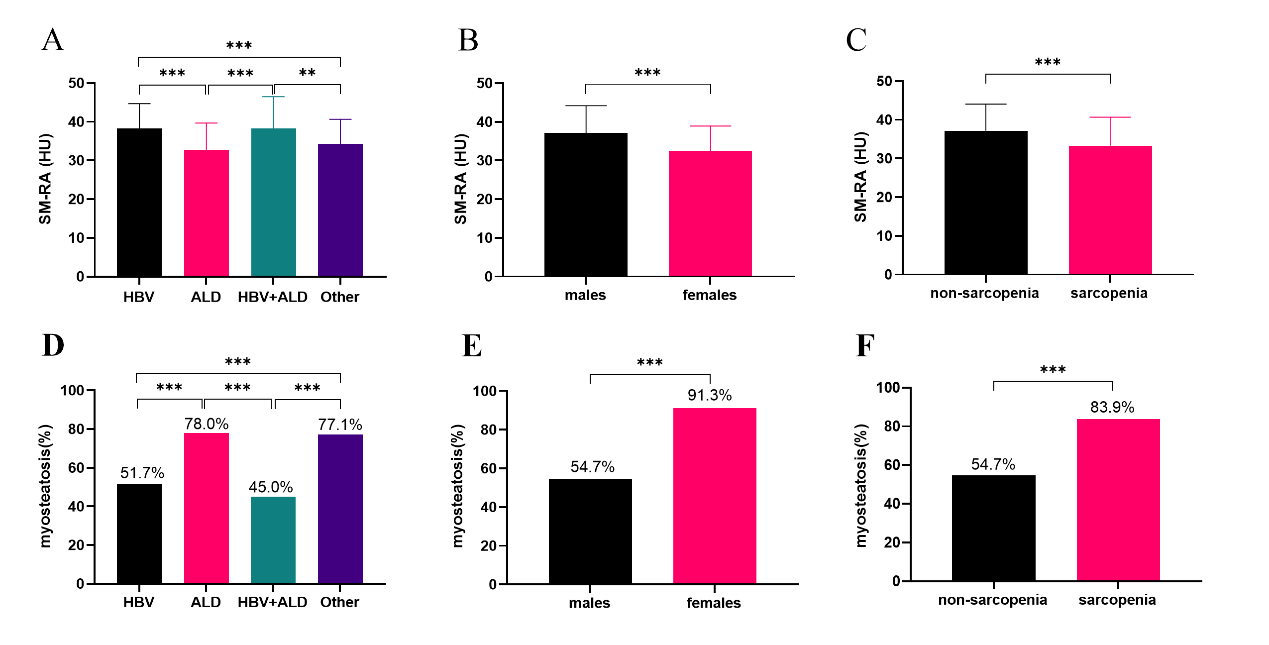


Supplementary Figure 2. Comparison of the SM-RA and incidence of myosteatosis by etiology [(A) and (D)], sex [(B) and (E)] and sarcopenia status [(C) and (F)].

Supplementary Table 1. Baseline characteristics of the cohort with male HBV-ACLF and classified by sarcopenia and myosteatosis

| Variables | Total  (n=201) | NN (n=102) | SN (n=9) | NM (n=70) | SM (n=20) | *P* value* |
| --- | --- | --- | --- | --- | --- | --- |
| Age (years) | 44 ± 10 | 41± 8 | 41± 11^&^ | 48± 9^#^ | 50± 10^#^ | <0.001 |
| BMI (kg/m^2^) | 24.3± 4.2 | 25.1± 4.0 | 21.7± 2.9^#^ | 24.3± 4.5^&^ | 20.8± 1.8^#^ | <0.001 |
| Liver cirrhosis, n (%) | 145 (72.1) | 68 (66.7) | 6 (66.7) | 54 (77.1) | 17 (85.0) | 0.243 |
| Ascites, n (%) | 151 (75.1) | 64 (62.7) | 6 (66.7) | 63 (90.0) ^#^ | 18 (90.0) | <0.001 |
| HE, n (%) | 53 (26.4) | 20 (19.6) | 2 (22.2) | 26 (37.1) | 5 (25.0) | 0.083 |
| AKI, n (%) | 13 (6.5) | 2 (2.0) | 0 | 6 (8.6) | 5 (25) ^#^ | 0.001 |
| Infection, n (%) | 157 (78.1) | 75 (73.5) | 8 (88.9) | 56 (80.0) | 18 (90.0) | 0.300 |
| Diabetes, n (%) | 37 (18.4) | 13 (12.7) | 3 (33.3) | 14 (20.0) | 7 (35.0) | 0.063 |
| MELD-Na score | 26.7± 8.2 | 24.3± 5.2 | 24.3± 5.5^&^ | 28.7± 9.0^#&^ | 33.3± 12.7^#^ | <0.001 |
| AARC score | 9 (2) | 9 (2) | 10 (2) | 9.5 (3) | 10 (2) | 0.128 |
| Number of OFs n (%) |  |  |  |  |  | 0.054 |
| 0 organ failure | 29 (14.4) | 20 (19.6) | 2 (22.2) | 4 (5.7) | 3 (15.0) |  |
| 1 organ failure | 96 (47.8) | 48 (47.1) | 6 (66.7) | 31 (44.3) | 11 (55.0) |  |
| ≥2 organ failures | 76 (37.8) | 34 (33.3) | 1 (11.1) | 35 (50.0) | 6 (30.0) |  |
| SM-RA | 39.2± 5.8 | 42.6± 4.4 | 43.1± 3.4^&^ | 34.9± 4.7^#^ | 35.1± 4.1^#^ | <0.001 |
| L3-SMI (cm^2^/m^2^) | 50.2± 8.1 | 53.3± 6.8 | 39.9± 2.1^#^ | 50.6± 6.7^#&^ | 37.7± 3.4^#^ | <0.001 |
| L3-VATI (cm^2^) | 39.4± 23.8 | 39.0± 22.0 | 28.5± 23.1 | 44.9± 25.8^&^ | 27.0± 20.6^#^ | 0.011 |
| L3-SATI (cm^2^) | 50.2± 30.3 | 52.5± 27.7 | 33.4± 16.0 | 55.0± 35.6^&^ | 28.7± 12.8^#^ | 0.001 |
| VSR | 0.83± 0.43 | 0.75± 0.33 | 0.80± 0.61 | 0.90± 0.48^#^ | 0.93± 0.54 | 0.082 |
| sarcopenia, n (%) | 29 (14.4) |  |  |  |  |  |
| Myosteatosis, n (%) | 90 (44.8) |  |  |  |  |  |
| Sarcopenic obesity, n (%) | 13 (6.5) |  |  |  |  |  |
| Visceral adiposity, n (%) | 115 (57.2) | 60 (58.8) | 3 (33.3) | 45 (64.3) | 7 (35.0) | 0.053 |

Continuous variables are shown as mean ± standard deviation or median (interquartile range) for normally and non-normally distributed continuous variables, respectively. Categorical variables are presented as numbers (percentages).

^#^ *P* < 0.05 vs patients with SN, NM or SM vs patients with NN.

^&^ *P* < 0.05 patients with SN or NM vs patients with SM.

* *P* value for difference among the four group

Supplementary Table 2. Study on the influence factors and prognosis of myosteatosis in patients with end-stage liver disease

| Author/ year | Country | Study population | Diagnosis method | Influence factors | Prognostic effects |
| --- | --- | --- | --- | --- | --- |
| Wang X et al. 2022^[1]^ | China | Decompensated liver cirrhosis  473 (males: females =235: 238) | Males: IMAC> -0.44  Females: IMAC> -0.37 | Advanced age, higher VSR, higher TATI | Myosteatosis was not an independent predictor of longer hospitalization |
| Ishizu Y et al. 2022^[2]^ | Japan | Liver cirrhosis  178 (males: females =92: 86) | BMI< 25kg/m^2^, SM-RA< 41HU; BMI≥25kg/m^2^, SM-RA< 33HU | Decreased L3-SMI and increased VATI were associated with myosteatosis progression | myosteatosis was independently associated with increased mortality |
| Ebadi M et al. 2022^[3]^ | Canada | Liver cirrhosis  855 (males: females=539: 86) | Below the lowest tertile of the study population：males: SM-RA< 33HU, females: SM-RA< 28HU | A significant linear, but weak correlation between SM-RA and L3-SMI (*r*=0.37) | Myosteatosis is associated with increased mortality, and the concomitant presence of myosteatosis and sarcopenia is associated with worse outcomes |
| Montano-Loza AJ et al. 2016^[4]^ | Canada | Liver cirrhosis  678 (males: females =457: 221) | BMI< 25kg/m^2^, SM-RA< 41HU; BMI≥25kg/m^2^, SM-RA< 33HU | Patients with myosteatosis had higher age, MELD, and Child-Pugh score, while had lower L3-SMI | Myosteatosis is an independent risk factor for increased 2-year mortality |
| Nardelli S et al. 2019^[5]^ | Italy | Liver cirrhosis  64 (males: females= =48: 16) | BMI< 25kg/m^2^, SM-RA< 41HU; BMI≥25kg/m^2^, SM-RA< 33HU | SM-RA showed a moderate positive correlation with L3-SMI (*r*=0.426) | Myosteatosis was an independent risk factor for overt hepatic encephalopathy in patients with liver cirrhosis |
| Bhanji RA et al. 2018^[6]^ | Canada | Liver cirrhosis  675 (males: females=454: 221） | BMI< 25kg/m^2^, SM-RA< 41HU; BMI≥25kg/m^2^, SM-RA< 33HU | Patients with myosteatosis were older with higher incidence rates in women | Myosteatosis was an independent risk factor for overt hepatic encephalopathy, but not an independent predictor for increased 2-year mortality |
| Masetti C et al. 2022^[7]^ | Italy | HCC Patients Undergoing Trans Arterial Embolization  151 (males: females=116: 35) | Males: IMAC> -0.44  Females: IMAC> -0.31 | Patients with myosteatosis had higher age and BMI; The incidence rate of myosteatosis is higher in men, alcohol or metabolic patients | Myosteatosis was not associated with length of hospitalization, complication rate, readmission and overall survival |
| Chen B-B et al. 2023^[8]^ | China | HCC receiving immunotherapy  138 (males: females=120: 18) | BMI< 25kg/m^2^, SM-RA< 41HU; BMI≥25kg/m^2^, SM-RA< 33HU | The patients with myosteatosis were older, and there was no difference in the incidence of sarcopenia | Myosteatosis was an independent predictor of poor progression-free survival and overall survival |
| Meister FA et al. 2022^[9]^ | Germany | HCC following Curative-Intent Surgery  100 (males: females=72: 28) | BMI< 25kg/m^2^, SM-RA< 41HU; BMI≥25kg/m^2^, SM-RA< 33HU | The incidence of myosteatosis is higher in patients with alcoholic liver disease | Myosteatosis was an independent risk factor for perioperative morbidity, but had no effect on long-term overall and recurrence-free survival |
| Shafaat O et al. 2023^[10]^ | The United States | Liver transplantation | BMI< 25kg/m^2^, SM-RA< 41HU; BMI≥25kg/m^2^, SM-RA< 33HU | NA | Sarcopenia was the factor significantly associated with post-LT mortality, while myosteatosis not |
| Meister FA et al. 2021^[11]^ | Germany | Liver transplantation  264 (males: females =141: 123) | Below the lowest quartile of the study population：  Male: SM-RA< 28.6 HU  females: SM-RA< 26.6 HU  males: IMAC> -0.35  females: IMAC> -0.32 | NA | Myosteatosis has a good predictive role on perioperative outcomes, and the predictive effect of myosteatosis diagnosed by SM-RA is better than that of IMAC |
| Czigany Z et  al. 2021^[12]^ | Germany | Liver transplantation  226 (males: females=117: 108) | BMI< 25kg/m^2^, SM-RA< 41HU; BMI≥25kg/m^2^, SM-RA< 33HU | NA | The prognostic value of myosteatosis seems to be more important in the early stage after liver transplantation |
| Czigany Z et al. 2020^[13]^ | Germany | Liver transplantation  226 (males: females =117: 108) | BMI< 25kg/m^2^, SM-RA< 41HU; BMI≥25kg/m^2^, SM-RA< 33HU | SM-RA is negatively correlated with MELD and Child-Pugh scores (*r*=-0.403, *r*=-0.338) | Myosteatosis is an independent risk factor for major complications within 90-days after liver transplantation; Muscle quality has a better predictive effect on short-term prognosis than muscle quantity |

**References**

[1] Wang X., Sun M., Li Y., Guo G., Yang W., Mao L.*, et al.* Association of myosteatosis with various body composition abnormalities and longer length of hospitalization in patients with decompensated cirrhosis[J]. Frontiers In Nutrition, 2022, 9: 921181.

[2] Ishizu Y., Ishigami M., Honda T., Imai N., Ito T., Yamamoto K.*, et al.* Factors associated with the progression of myosteatosis in patients with cirrhosis[J]. Nutrition (Burbank, Los Angeles County, Calif.), 2022, 103-104: 111777.

[3] Ebadi M., Tsien C., Bhanji R.A., Dunichand-Hoedl A.R., Rider E., Motamedrad M.*, et al.* Skeletal Muscle Pathological Fat Infiltration (Myosteatosis) Is Associated with Higher Mortality in Patients with Cirrhosis[J]. Cells, 2022, 11(8) :1345.

[4] Montano-Loza A.J., Angulo P., Meza-Junco J., Prado C.M.M., Sawyer M.B., Beaumont C.*, et al.* Sarcopenic obesity and myosteatosis are associated with higher mortality in patients with cirrhosis[J]. Journal of Cachexia, Sarcopenia and Muscle, 2016, 7(2): 126-135.

[5] Nardelli S., Lattanzi B., Merli M., Farcomeni A., Gioia S., Ridola L.*, et al.* Muscle Alterations Are Associated With Minimal and Overt Hepatic Encephalopathy in Patients With Liver Cirrhosis[J]. Hepatology (Baltimore, Md.), 2019, 70(5): 1704-1713.

[6] Bhanji R.A., Moctezuma-Velazquez C., Duarte-Rojo A., Ebadi M., Ghosh S., Rose C.*, et al.* Myosteatosis and sarcopenia are associated with hepatic encephalopathy in patients with cirrhosis[J]. Hepatology International, 2018, 12(4): 377-386.

[7] Masetti C., Pugliese N., Lofino L., Colapietro F., Ceriani R., Lleo A.*, et al.* Myosteatosis Is Not Associated with Complications or Survival in HCC Patients Undergoing Trans Arterial Embolization[J]. Journal of Clinical Medicine, 2022, 12(1) :262.

[8] Chen B.-B., Liang P.-C., Shih T.T.-F., Liu T.-H., Shen Y.-C., Lu L.-C.*, et al.* Sarcopenia and myosteatosis are associated with survival in patients receiving immunotherapy for advanced hepatocellular carcinoma[J]. European Radiology, 2023, 33(1): 512-522.

[9] Meister F.A., Lurje G., Verhoeven S., Wiltberger G., Heij L., Liu W.-J.*, et al.* The Role of Sarcopenia and Myosteatosis in Short- and Long-Term Outcomes Following Curative-Intent Surgery for Hepatocellular Carcinoma in a European Cohort[J]. Cancers, 2022, 14(3) :720.

[10] Shafaat O., Liu Y., Jackson K.R., Motter J.D., Boyarsky B.J., Latif M.A.*, et al.* Association between Abdominal CT Measurements of Body Composition before Deceased Donor Liver Transplant with Posttransplant Outcomes[J]. Radiology, 2023, 306(3): e212403.

[11] Meister F.A., Bednarsch J., Amygdalos I., Boecker J., Strnad P., Bruners P.*, et al.* Various myosteatosis selection criteria and their value in the assessment of short- and long-term outcomes following liver transplantation[J]. Scientific Reports, 2021, 11(1): 13368.

[12] Czigany Z., Kramp W., Lurje I., Miller H., Bednarsch J., Lang S.A.*, et al.* The role of recipient myosteatosis in graft and patient survival after deceased donor liver transplantation[J]. Journal of Cachexia, Sarcopenia and Muscle, 2021, 12(2): 358-367.

[13] Czigany Z., Kramp W., Bednarsch J., van der Kroft G., Boecker J., Strnad P.*, et al.* Myosteatosis to predict inferior perioperative outcome in patients undergoing orthotopic liver transplantation[J]. American Journal of Transplantation : Official Journal of the American Society of Transplantation and the American Society of Transplant Surgeons, 2020, 20(2): 493-503.
